# Outcomes of Chylothorax Nonoperative Management After Cardiothoracic Surgery: A Systematic Review and Meta-Analysis

**DOI:** 10.21470/1678-9741-2022-0326

**Published:** 2023-09-19

**Authors:** Laura Lucato dos Santos, Clara Lucato dos Santos, Natasha Kasakevic Tsan Hu, Leticia Nogueira Datrino, Guilherme Tavares, Luca Schiliró Tristão, Marina Feliciano Orlandini, Maria Carolina Andrade Serafim, Francisco Tustumi

**Affiliations:** 1 Departament of Gastroenterology, Faculdade de Medicina, Universidade de São Paulo, São Paulo, São Paulo, Brazil; 2 Departament of Evidence-Based Medicine, Faculdade de Medicina, Centro Universitário Lusíada, Santos, São Paulo, Brazil; 3 Departament of Evidence-Based Medicine, Oya Care, São Paulo, São Paulo, Brazil; 4 Departament of Health Sciences, Faculdade de Medicina, Hospital Israelita Albert Einstein, São Paulo, São Paulo, Brazil

**Keywords:** Chylothorax, Lymphatic System, Thoracic Duct, Thoracic Surgery, Morbidity, Reoperation, Cardiac Arrhythmias

## Abstract

**Introduction:**

Chylothorax after thoracic surgery is a severe complication with high morbidity and mortality rate of 0.10 (95% confidence interval [CI] 0.06 – 0.02). There is no agreement on whether nonoperative treatment or early reoperation should be the initial intervention. This systematic review and meta-analysis aimed to evaluate the outcomes of the conservative approach to treat chyle leakage after cardiothoracic surgeries.

**Methods:**

A systematic review was conducted in PubMed®, Embase, Cochrane Library Central, and LILACS (Biblioteca Virtual em Saúde) databases; a manual search of references was also done. The inclusion criteria were patients who underwent cardiothoracic surgery, patients who received any nonoperative treatment (*e.g.*, total parenteral nutrition, low-fat diet, medium chain triglycerides), and studies that evaluated chylothorax resolution, length of hospital stay, postoperative complications, infection, morbidity, and mortality.

**Central Message:**

Nonoperative treatment for chylothorax after cardiothoracic procedures has significant hospital stay, morbidity, mortality, and reoperation rates.

**Results:**

Twenty-two articles were selected. Pulmonary complications, infections, and arrhythmia were the most common complications after surgical procedures. The incidence of chylothorax in cardiothoracic surgery was 1.8% (95% CI 1.7 – 2%). The mean time of maintenance of the chest tube was 16.08 days (95% CI 12.54 – 19.63), and the length of hospital stay was 23.74 days (95% CI 16.08 – 31.42) in patients with chylothorax receiving nonoperative treatment. Among patients that received conservative treatment, the morbidity event was 0.40 (95% CI 0.23 – 0.59), and reoperation rate was 0.37 (95% CI 0.27 – 0.49). Mortality rate was 0.10 (95% CI 0.06 – 0.02).

**Conclusion:**

Nonoperative treatment for chylothorax after cardiothoracic procedures has significant hospital stay, morbidity, mortality, and reoperation rates.

## INTRODUCTION

Chyle is an opaque, milky-white fluid consisting mainly of emulsified fats that pass through the lacteals of the small intestines into the lymphatic system^[1]^. This fluid contains lipids, proteins, immunoglobulins, lymphocytes, vitamins, and electrolytes^[2]^. Chyle leak is a potentially devastating phenomenon and may impair nutrition, compromise and delay wound healing, and prolong hospitalization^[3]^.

Postoperative chylothorax is usually caused by injuries to the thoracic duct or to its tributaries during surgery^[4]^. Chylothorax may happen in several types of cardiothoracic surgery, including esophagectomy, lobectomy, cardiac procedures, and mediastinal tumors resection^[5,6,7,8,9]^. The diagnosis of chylothorax consists of evaluating triglyceride levels, cholesterol values, and microscopy crystals^[10]^.

Reoperation with thoracic duct ligation, with direct closure of the ruptured lymph vessel or with thoracic duct obliteration, is one of the treatment choices for this complication^[11,12]^. Other therapeutic approaches to treat chylothorax comprise lymphangiography with thoracic duct embolization^[13]^. However, nonoperative management of postoperative chylothorax (NMPC) is usually considered the first approach, and it is a non-invasive strategy based on prolonged fasting or a low-fat diet. The central idea is to reduce the lymphatic system content to progressively lower the lymphatic leak flow^[14]^. NMPC comprises total parenteral nutrition (TPN) and oral or enteral medium-chain triglycerides (MCT)^[15]^.

Currently, there is no scientific consensus regarding the optimal management of chylothorax after cardiothoracic surgeries. Consequently, the present review aims to evaluate the outcomes of conservative management of postoperative chylothorax.

## METHODS

### Protocol Register

This systematic review and meta-analysis was submitted to the International Prospective Register of Systematic Reviews (or PROSPERO)^[16]^ under the trial registry CRD42021235243. Search strategy and selection articles were based on the Preferred Reporting Items for Systematic Reviews and Meta-Analysis (or PRISMA) guideline^[17]^.

### Search and Selection

Two researchers carried out, independently, the search and selection of the evidence in the following scientific databases: PubMed®, Embase, Cochrane, and LILACS (Biblioteca Virtual em Saúde); manual search evaluating the references of primary studies and other reviews was done. The selection was completed in July 2022. The search strategy on MEDLINE® was: (Lymphatic fistula OR Lymphatic leak OR Lymphatic fistulae OR Chyle leak OR Chylous ascites OR Chyloperitoneum OR Chylous Peritonitis OR Chylothorax OR Thoracic duct OR Duct, Thoracic OR Cisterna Chyli OR Cisterna Chylus OR Lymphatic vessels OR Lymphatic Venule) AND (Diet, fat restricted OR Diet low fat OR Diet Fat Free) AND (Nutrition, Parenteral OR Parenteral Feeding OR Intravenous Feeding). Similar terms were used in the other databases.

### Eligibility

The eligibility criteria were: (1) patients who underwent cardiothoracic surgery; (2) patients who received any conservative treatment (*e.g.*, TPN, low-fat diet, MCT) or surgical treatment; (3) studies that evaluate chylothorax, postoperative complications, infection, morbidity, and mortality; (4) studies in English or Portuguese; (5) clinical trial or observational studies (prospective or retrospective).

### Data Extraction

The following data were extracted from the studies: (1) general information (authors, year of publication, study design); (2) patients and chylothorax specifications (total patients, cardiothoracic procedure, patients with chylothorax, chylothorax definition, and mean age); (3) conservative treatment; (4) variables related with population and outcomes (chest time maintenance, reoperation, morbidity, complications, length of hospital stay, mortality).

### Risk of Bias and Certainty Assessment

The articles were assessed for bias risk using the Risk of Bias in Non-Randomized Studies of Intervention (ROBINS-I)^[18]^ assessment tool. Grading of Recommendations, Assessment, Development and Evaluations (GRADE) (https://www.gradepro.org/)^[19]^ was used to evaluate the quality of the evidence.

### Synthesis and Statistical Analysis

The authors extracted and analyzed the absolute numbers for each outcome using the software Comprehensive Meta-Analysis. The measures used to express benefit and harm varied according to the outcomes and were expressed by continuous variables (mean and standard deviation [SD]) or by categorical variables (absolute number of events). In continuous measures, the results were mean diference and SD. The results were synthesized in a meta-analysis. The heterogeneity of effect sizes among studies was assessed with I^2^ statistics. Pooled-effect measures were calculated with 95% confidence interval (CI), and the significance level used was 0.05.

## RESULTS

### Baseline Characteristics of the Included Studies

**Fig. 1:**
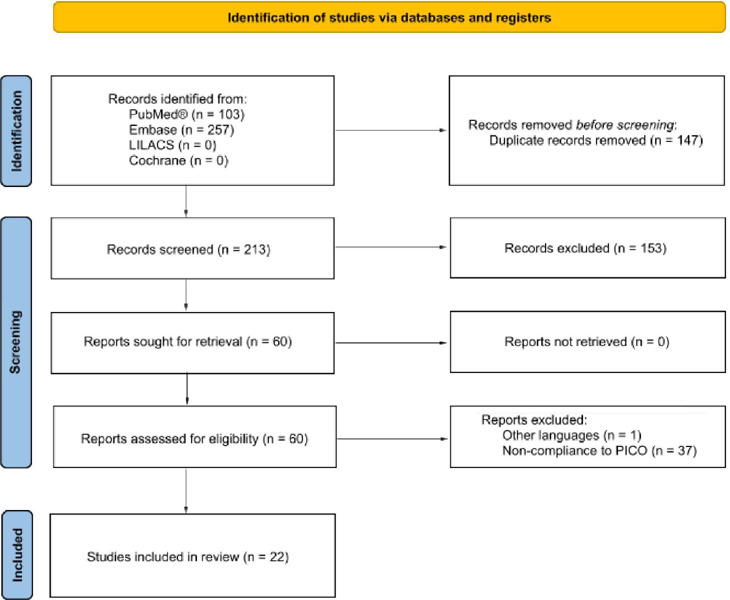
Preferred Reporting Items for Systematic Reviews and Meta-Analysis (PRISMA) flow diagram. PICO=Patient, intervention, comparison, or outcome.

After applying eligibility criteria, 22 studies were selected for qualitative and quantitative analysis^[14,20,21,22,23,24,25,26,27,28,29,30,31,32,33,34,35,36,37,38,39,40]^. The selection flow diagram is shown in Figure 1. Included studies comprised 497 patients with chylothorax, with a mean age of 50.19 years old. Baseline characteristics of the included studies are reported in Table 1.

**Table 1:** Baseline characteristics of the included studies

Autor	Year	Design	Total patients	Cardiothoracic surgery	Chylothorax (n)	Chylothorax definition	Mean age (years)	Nonoperative treatment	Reoperation method
Guillem et al^[29]^	2004	Cohort	Uninformed	Esophagectomy, lobectomy, gastrectomy	8	Daily output of at least 250 ml or chyle leaks with a duration of at least 7 days	52	TPN + MCT	Duct ligation
Marts et al^[32]^	1992	Cohort	Uninformed	Congenital heart surgery, esophagectomy, trauma, miscellaneous thoracic procedures	29	Milky-appearing fluid, a pH between 7.4 and 7.8, triglyceride level > 110 mg/dL, fat globules seen on a Sudan III stain, or chylomicrons proven by electrophoresis. In addition, a specific gravity > 1.012 or a high pleural fluid cell count with lymphocyte predominance	20	TPN + low-fat diet; low-fat diet + MCT	Duct ligation
Alexiou et al^[20]^	1998	Cohort	523	Esophagectomy	21	Confirmed by the change in fluid character to milky after commencement of enteral feeding and the presence of chylomicrons on biochemical analysis of the pleural fluid	65	TPN	Duct ligation
Allaham et al^[21]^	2006	Cohort	1159	Aortic surgical procedures	5	Triglyceride levels 100 mg/dL or predominant presence of lymphocytes confirmed the diagnosis	64	TPN	Duct ligation
Bolger et al^[22]^	1991	Cohort	537	Esophagectomy	11	Drainage of straw-coloured fluid from the chest drain continued for > 5 days and it was confirmed as a chylous leak by its milky white appearance following the administration of cream through the nasogastric tube	Uninformed	TPN	Duct ligation
Bonavina et al^[23]^	2001	Cohort	316	Esophagectomy	3	Presence of milky fluid in the chest tube and bilateral pleural effusion after the removal of the chest tube	56 to 63	TPN	Duct ligation
Cerfolio et al^[24]^	1996	Cohort	11315	Esophagectomy, aortic surgical procedures, pulmonary resections, mediastinal mass resection	47	Triglyceride content of 110 mg/dl or greater and the presence of chylomicrons in the pleural fluid in all patients	65	TPN;MCT	Duct ligation
Dugue et al^[26]^	1998	Cohort	850	Esophagectomy	23	Suspected as early as the third postoperative day when the chest drainage output was > 500 ml per 24 h with a lymphocyte count of > 50%. The diagnosis was confirmed by injection of a cream rich diet through the nasogastric tube which resulted in a milky appearance of the pleural fluid	54	TPN	Duct ligation
Lagarde et al^[30]^	2005	Cohort	536	Esophagectomy	20	Drain output changed from yellow to milky after start of enteral feeding (or administration of cream) and changed back again to yellow after discontinuation of enteral feeding. Triglyceride concentration in the drain output was > 1.2 mmol/L	62	TPN	Duct ligation
Merigliano et al^[33]^	2000	Cohort	1787	Esophagectomy	11	Suspected in the presence of excessive (> 1000 mL per day) chest or mediastinal output continuing for >2 days and it was confirmed by physical and biochemical analysis of the fluid	57	TPN	Duct ligation
Seow et al^[35]^	2010	Cohort	442	Esophagectomy	10	Postoperative lymph leak > 500 ml over 48 h	Uninformed	TPN	Duct ligation
Shah et al^[36]^	2012	Cohort	892	Esophagectomy	34	Change in the quality of chest tube drainage to milky white drainage, regardless of chest tube output, or confirmation of chylomicrons or triglycerides in the pleural drainage in patients with high-volume drainage	67	TPN; enteral nutrition	Duct ligation
Shen et al^[37]^	2014	Cohort	344	Esophagectomy	10	Laboratory confirmation of elevated triglycerides (> 110 mL/dL) or positive Sudan III stain in the setting of sustained drainage	Uninformed	TPN	Duct ligation
Petrella et al^[14]^	2020	Cohort	5072	Esophagectomy, pulmonary resections, mediastinal mass resection	30	Chylous leakage from the chest drainage with the presence of triglycerides (> 110mg/dL) in the pleural fluid	63	TPN;TPN + low-fat diet	Duct ligation
Furukawa et al^[28]^	2018	Cohort	818	Pulmonary resection	14	Uninformed	Uninformed	TPN; low-fat diet	Uninformed
Takuwa et al^[39]^	2013	Cohort	1580	Pulmonary resection	37	Chylous leakage from a chest tube with an elevated triglyceride level (> 110mg/dL) in the drainage fluid	69	TPN + low-fat diet	Duct ligation
Pego-Fernandes et al^[34]^	2011	Cohort	3092	Cardiac surgery	64	High level of triglycerides (> 110 mg/dL) or a level of triglyceride/cholesterol > 1 in the pleural fluid; presence of leukocytes and chylomicrons in the fluid	2	TPN;TPN + low-fat diet; MCT	Duct ligation
Chan et al^[25]^	2005	Cohort	1257	Cardiac surgery	48	Triglyceride levels in pleural fluid had to be 1.2 mmol/L, with a total cell number 1,000 cell s/mL and a predominance of mononuclear cells	1	TPN; low-fat diet	Duct ligation
Fahimi et al^[27]^	2001	Cohort	Uninformed	Aortic surgery, pulmonary resection, cardiac surgery, mediastinoscopy, sypathectomy	12	Postoperative pleural or epicardial effusion unexpectedly large and presence of triglycerides and chylomicrons in the fluid	61	MCT	Duct ligation; fibrin glue if site of injury could not be identified
Le Pimpec-Barthes et al^[31]^	2002	Cohort	Uninformed	Pulmonary resection	26	Appearance of a milky pleural effusion with an elevated triglyceride level > 100 mg/dL (triglycerides 1 mg/100 mL = 0.0113 mmol/L), a lymphocyte count > 90% of total white blood cell count, and total protein concentration approaching that of plasma	57	TPN;TPN + MCT	Duct ligation; suture of leaking collateral; fibrin glue
Shimizu et al^[38]^	2002	Cohort	1110	Pulmonary resection	26	Presence of triglycerides (> 110 mg/dL)and chylomicrons in the drainage fluid	62	TPN	Uninformed
Worthington et al^[40]^	1995	Cohort	Uninformed	Penetrating chest trauma	8	Uninformed	23	TPN; MCT	Duct ligation

MCT=medium-chain triglycerides;TPN=total parenteral nutrition

The cardiothoracic procedures performed included: esophagectomy, lobectomy, gastrectomy, congenital heart surgery, trauma treatment, miscellaneous thoracic procedure, aortic surgical procedure, pulmonary resection, mediastinal mass resection, cardiac surgery, mediastinoscopy, and sympathectomy.

### Chylothorax Incidence

Seventeen studies analyzed this outcome. The chylothorax incidence in patients that underwent cardiothoracic surgery was 1.8% (rate: 0.018; 95% CI: 0.017 – 0.020) (Figure 2).

**Fig. 2:** Chylothorax incidence after cardiothoracic surgery. CI=confdence interval.

Study name	Statistics for each study					Event rate and 95% CI
	Event rate	Lower limit	Upper limit	Z-Value	p-Value	
Alexiou et al	0,040	0,026	0,061	-14,250	0,000	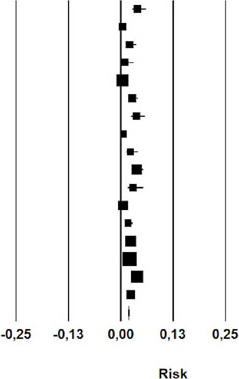
Allaham et al	0,004	0.002	0,010	-12,141	0.000	
Bolger et al	0,020	0,011	0,037	-12,695	0,000	
Bonavina et al	0,009	0,003	0,029	-8,012	0.000	
Cerfolio et al	0,004	0,003	0,006	-37,488	0,000	
Dugue et al	0,027	0,018	0,040	-16,946	0,000	
Lagarde et al	0,037	0,024	0,057	-14,262	0,000	
Merigliano et al	0,006	0,003	0,011	-16,810	0,000	
Seow et al	0,023	0,012	0,042	-11,773	0,000	
Shah et al	0,038	0,027	0,053	-18,461	0,000	
Shen et al	0,029	0,016	0,053	-10,933	0,000	
Petrella et al	0,006	0,004	0,008	-27,984	0,000	
Furukawa et al	0,017	0,010	0,029	-15,025	0,000	
Takuwa et al	0,023	0,017	0,032	-22,425	0,000	
Pego-Fernandes et al	0,021	0,016	0,026	-30,533	0,000	
Chan et al	0,038	0,029	0,050	-21,922	0,000	
Shimizu et al	0,023	0,016	0,034	-18,797	0,000	
	0,018	0,017	0,020	-79,890	0,000	

### Complications

The most common complications in patients undergoing nonoperative management of chylothorax were pulmonary complications (respiratory failure and pneumonia), infections, and arrhythmia. Other complications after surgical procedure comprised urinary tract infection, the necessity of prolonged ventilation, prolonged air leak, cervical anastomotic leak, reintubation, renal failure, sepsis, empyema, acute hemorrhagic pseudocyst, delirium, mediastinal chyloma, atelectasis, and seizure.

### Chest Tube

Twelve studies analyzed the length of chest tube usage in patients undergoing nonoperative management of chylothorax. The mean time of chest tube maintenance was 16.08 days (95% CI 12.54 – 19.63) (Figure 3).

**Fig. 3:** Chest tube time duration after initial nonoperative management of postoperative chylothorax. CI=confdence interval.

Study name	Statistics for each study							Event rate and 95% CI
	Mean	Standard error	Variance	Lower limit	Upper limit	Z-Value	p-Value	
Guillem et al	25,300	4,950	24,500	15,599	35,001	5,111	0,000	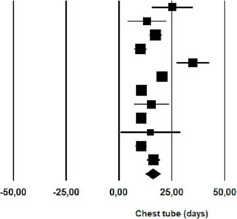
Marts et al	13,300	4,642	21,552	4,201	22,399	2,865	0,004	
Alexiou et al	17,300	1,528	2,333	14 306	20,294	11,326	0,000	
Allaham et al	10,200	1,565	2,450	7,13?	13,268	6,517	0,000	
Bolger et al	35,000	3,920	15,364	27,318	42,682	8,929	0,000	
Merigliano et al	20,500	0,603	0,364	19,318	21,682	33,995	0,000	
Shah et al	10,700	1,200	1,441	8,347	13,053	8,913	0,000	
Furukawa et al	15,500	4,276	18,286	7,119	23,881	3,625	0,000	
Takuwa et al	10,500	1,200	1,440	8,148	12,852	8,749	0,000	
Chan et al	15,000	7,217	52,083	0,855	29,145	2,078	0,038	
Le Pimpec-Barthes et al	10,500	1,451	2,106	7,656	13,344	7,235	0,000	
Worthington et al	16,400	1,556	2,420	13,351	19,449	10,542	0,000	
	16,082	1,808	3,270	12,538	19,626	8,894	0,000	

### Length of Stay

The mean length of hospital stay was 23.74 days (95% CI 16.08 – 31.42) for patients undergoing nonoperative management of chylothorax after cardiothoracic procedures (Figure 4).

**Fig. 4:** Length of hospital stay (LOS) after initial nonoperative management of postoperative chylothorax. CI=confdence interval.

Study name	Statistics for each study							Event rate and 95% CI
	Mean	Standard error	Variance	Lower limit	Upper limit	Z-Value	p-Value	
Guillem et al	32,000	6,718	45,125	18,834	45,166	4,764	0,000	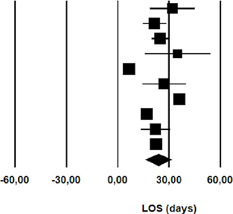
Marts et al	21,500	3,528	12,448	14,585	28,415	6,094	0,000	
Alexiou et al	24,800	2,510	6,298	19,881	29,719	9,882	0,000	
Allaham et al	35,000	9,839	96,800	15,717	54,283	3,557	0,000	
Bonavina et al	6,700	1,443	2.083	3,871	9,529	4,642	0,000	
Lagarde et al	27,000	6,485	42.050	14,290	39,710	4,164	0,000	
Merigliano et al	36,000	1,146	1,313	33,754	38,246	31,421	0,000	
Shah et al	17,000	1,200	1,441	14,647	19,353	14,161	0,000	
Chan et al	22,000	4,474	20,021	13,230	30,770	4,917	0,000	
Worthington et al	22,400	1,768	3,125	18,935	25,865	12,671	0,000	
	23,745	3,913	15,314	16,075	31,415	6,068	0,000	

### Morbidity

The morbidity among patients that received nonoperative treatment was 39.7% (rate: 0.397; 95% CI 0.23 – 0.59) (Figure 5).

**Fig. 5:** Morbidity after initial nonoperative management of postoperative chylothorax. CI=confdence interval.

Study name	Statistics for each study					Event rate and 95% CI
	Event rate	Lower limit	Upper limit	Z-Value	p-Value	
Alexiou et al	0,429	0,240	0,640	-0,652	0,514	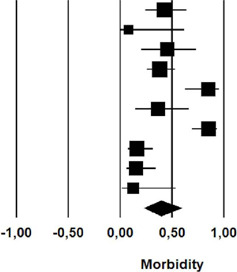
Allaham et al	0,083	0,005	0,622	-1,623	0,105	
Bolger et al	0,455	0,203	0,732	-0,301	0,763	
Cerfolio et al	0,383	0,256	0,528	-1,589	0,112	
Lagarde et al	0,850	0,624	0,951	2,770	0,006	
Merigliano et al	0,364	0,143	0,661	-0,893	0,372	
Shah et al	0,853	0,692	0,937	3,630	0,000	
Takuwa et al	0,162	0,075	0,317	-3,682	0,000	
Shimizu et al	0,154	0,059	0,345	-3,136	0,002	
Worthington et al	0,125	0,017	0,537	-1,820	0,069	
	0,397	0,228	0,594	-1,028	0,304	

### Mortality

The mortality was 9.9% in patients undergoing nonoperative management of chylothorax (rate: 0.099; 95% CI 0.06 – 0.02) (Figure 6).

**Fig. 6:** Mortality after initial nonoperative management of postoperative chylothorax. CI=confdence interval.

Study name	Statistics for each study					Event rate and 95% CI
	Event rate	Lower limit	Upper limit	Z-Value	p-Value	
Guillem et al	0,056	0,003	0,505	-1,947	0,052	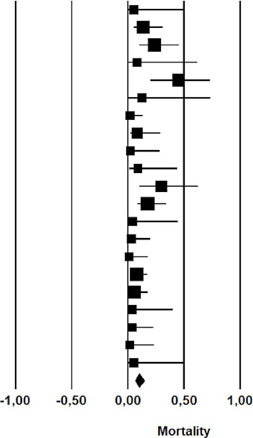
Marts et al	0,138	0.053	0,315	-3,403	0,001	
Alexiou et al	0,238	0,103	0,460	-2,270	0,023	
Allaham et al	0,083	0,005	0,622	-1,623	0,105	
Bolger et al	0,455	0,203	0,732	-0,301	0,763	
Bonavina et al	0,125	0,007	0,734	-1,287	0,198	
Cerfolio et al	0,021	0,003	0,136	-3,788	0,000	
Dugue et al	0,087	0,022	0,289	-3,177	0,001	
Lagarde et al	0,024	0,001	0,287	-2,594	0,009	
Merigliano et al	0,091	0,013	0,439	-2,195	0,028	
Seow et al	0,300	0,100	0,624	-1,228	0,220	
Shah et al	0,176	0,081	0,341	-3,424	0,001	
Shen et al	0,045	0,003	0,448	-2,103	0,035	
Petrella et al	0,033	0,005	0,202	-3,311	0,001	
Takuwa et al	0,013	0,001	0,178	-3,033	0,002	
Pego-Fernandes et al	0,078	0,033	0,174	-5,299	0,000	
Chan et al	0,063	0,020	0,177	-4,542	0,000	
Fahimi et al	0,038	0,002	0,403	-2,232	0,026	
Le Pimpec-Barthes et al	0,038	0,005	0,228	-3,156	0,002	
Shimizu et al	0,019	0,001	0,236	-2,781	0,005	
Worthington et al	0,056	0,003	0,505	-1,947	0,052	
	0,099	0,064	0,150	-9,177	0,000	

### Reoperation

Among patients with chylothorax that received initial nonoperative management of chylothorax, 37.1% (rate: 0.371; 95% CI: 0.270 – 0.486) required reoperation with thoracic duct ligation (Figure 7).

**Fig. 7:** Reoperation rate after initial nonoperative management of postoperative chylothorax. CI=confdence interval.

Study name	Statistics for each study					Event rate and 95% CI
	Event rate	Lower limit	Upper limit	Z-Value	p-Value	
Guillem et al	0,375	0,125	0,715	-0,699	0,484	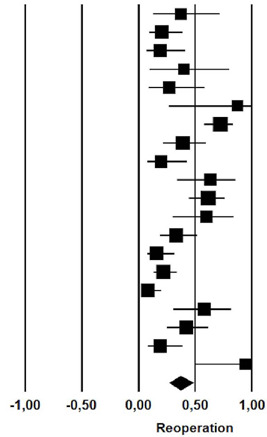
Marts et al	0,207	0,096	0,390	-2,931	0,003	
Alexiou et al	0,190	0,073	0,412	-2,604	0,009	
Allaham et al	0,400	0,100	0,800	-0,444	0,657	
Bolger et al	0,273	0,090	0,586	-1,449	0,147	
Bonavina et al	0,875	0,266	0,993	1,287	0,198	
Cerfolio et al	0,723	0,580	0,832	2,948	0.003	
Dugue et al	0,391	0,218	0,598	-1,034	0,301	
Lagarde et al	0,200	0,077	0,428	-2,480	0,013	
Merigliano et al	0,636	0,339	0,857	0,893	0,372	
Shah et al	0,618	0,447	0,763	1,359	0,174	
Shen et al	0,600	0,297	0,842	0,628	0,530	
Petrella et al	0,333	0,190	0,516	-1,790	0,074	
Takuwa et al	0,162	0,075	0,317	-3,682	0,000	
Pego-Fernandes et al	0,219	0,134	0,336	-4,210	0,000	
Chan et al	0,083	0,032	0,202	-4,592	0,000	
Fahlmi et al	0,583	0,308	0,815	0,575	0,566	
Le Pimpec-Barthes et al 0,423	0,252	0,615	-0,781	0,435		
Shimizu et al	0,192	0,082	0,387	-2,884	0.004	
Worthington et al	0,944	0,495	0,997	1,947	0,052	
	0,371	0,270	0,486	-2,201	0,028	

### Risk of Bias and Certainty Assessment

The GRADE critical appraisal showed that most outcomes presented low or very low certainty assessment. The main reasons for the reduced certainty were due to risk of selection bias, clinical heterogeneity among studies (comprising a variety of surgical procedures), and imprecision of data synthesis for some outcomes (Supplementary File 1). ROBINS-I tool showed that the main concerns were risk for selection bias and classification of the intervention (Supplementary File 2).

**Supp. File 1:** Certainty assessment.

Certainty assessment						
Studies	Risk of bias	Inconsistency	Indirectness	Imprecision	Publication bias	Overall certainty of evidence
**Chylothorax incidence in cardiothoracic surgery**						
17 observational studies	Serious^a^	Serious^b^	Not serious	Not serious	None	⊕⊕
						Low
**Length of chest tube usage**						
12 observational studies	Serious^a^	Serious^b^	Not serious	Not serious	None	⊕⊕
						Low
**Morbidity**						
10 observational studies	Serious^a^	Serious^b^	Not serious	Very serious^c^	None	⊕
						Very low
**Mortality**						
21 observational studies	Serious^a^	Serious^b^	Not serious	not Serious	None	⊕⊕
						Low
**Reoperation**						
20 observational studies	Serious^a^	Serious^b^	Not serious	Serious^d^	None	⊕
						Very low
**Length of hospital stay**						
10 observational studies	Serious^a^	Serious^b^	Not serious^e^	Very serious^e^	None	⊕
						Very low

^a^Risk for selection bias; ^b^Signifcant clinical heterogeneity; ^c^95% CI range > 30%; ^d^95% CI range > 15% and ≤ 30%; ^e^95% CI range > 15 days CI=confdence interval

**Supp. File 2:** Risk of bias assessment.

	1. Bias dueto confounding	2. Bias in selection of participants into the study	3. Bias in classification of interventions	4. Bias due to deviations from intended interventions	5. Bias due to missing data	6. Bias in measurement of outcomes	7. Bias in selection of the reported results	8. Overall bias
Guillem et al.	Low	Critical	Moderate	Low	Moderate	Low	Low	Moderate
Marts et al.	Low	Critical	Moderate	Low	Moderate	Low	Low	Moderate
Alexiou et al.	Low	Critical	Moderate	Low	Low	Low	Low	Moderate
Allaham et al.	Low	Critical	Moderate	Low	Low	Low	Low	Moderate
Bolger et al.	Low	Critical	Moderate	Low	Moderate	Low	Low	Moderate
Bonavina et al.	Low	Critical	Moderate	Low	Low	Low	Low	Moderate
Cerfolio et al.	Low	Critical	Moderate	Low	Low	Low	Low	Moderate
Dugue et al.	Low	Critical	Moderate	Low	Low	Low	Low	Moderate
Lagarde et al.	Low	Critical	Moderate	Low	Low	Low	Low	Moderate
Merigliano et al.	Low	Critical	Moderate	Low	Low	Low	Low	Moderate
Seow et al.	Low	Critical	Moderate	Low	Moderate	Low	Low	Moderate
Shah et al.	Low	Critical	Moderate	Low	Low	Low	Low	Moderate
Shen et al.	Low	Critical	Moderate	Low	Moderate	Low	Low	Moderate
Petrella et al.	Low	Critical	Moderate	Low	Low	Low	Low	Moderate
Furukawa et al.	Low	Critical	Critical	Low	Serious	Low	Low	Serious
Takuwa et al.	Low	Critical	Moderate	Low	Low	Low	Low	Moderate
Pego-Fernandes et al.	Low	Critical	Moderate	Low	Low	Low	Low	Moderate
Chan et al.	Low	Critical	Moderate	Low	Low	Low	Low	Moderate
Fahimi et al.	Low	Critical	Moderate	Low	Moderate	Low	Low	Moderate
Le Pimpec-Barthes et al.	Low	Critical	Moderate	Low	Moderate	Low	Low	Moderate
Shimizu et al.	Low	Critical	Moderate	Low	Low	Low	Low	Moderate
Worthington et al.	Low	Critical	Critical	Low	Moderate	Low	Low	Serious

The Risk of Bias in Non-Randomized Studies of Interventions (ROBINS-I) assessment tool for cohort-type studies.

## DISCUSSION

NMPC as the first approach strategy for chylothorax is associated with a high risk for morbidity and mortality, with prolonged hospital stay and time of chest tube. More than one-third of the patients undergoing nonoperative management will require reoperation.

Since chylothorax is a rare complication after cardiothoracic surger y (incidence rate = 1.8%), trials comparing the treatment options with a satisfactory sample size are difficult to be performed. There is no consensus on the time required for the decision to operate on the patient after a failing nonoperative initial management^[14,20]^. Consequently, it is impossible to provide the highest standard of evidence-based recommendation for any treatment approach. However, considering the high morbidity and mortality, length of hospital stay, and demand for reintervention, it is reasonable to consider early reoperation after a chylothorax diagnosis in postoperative cardiothoracic procedures. Only future studies that compare nonoperative methods and early invasive intervention for the management of chylothorax will allow a definitive answer. Merigliano et al.^[33]^ assessed chylothorax outcomes after esophagectomy and advocated for early reoperation with thoracic duct ligation. The authors found high morbidity with a high rate of demand for reoperation after initial treatment with TPN without oral diet intake. Besides, no reliable predictive variables for the success of the nonoperative management were found. Wemyss‐Holden et al.^[41]^ also defend an aggressive early intervention for postoperative chylothorax within 48 hours from the diagnosis. The idea is to act as early as the patient remains relatively fit, without nutritional and immunological debilitation. Besides, early reoperation decisions allow low adherence and better tissue visualization, facilitating direct closure of the thoracic duct injury^[42]^.

Prolonged and constant chyle drainage through the chest tube will lead patients to nutritional deficit and immunological depletion, which will make them vulnerable to hospital-acquired infections^[43]^. The chyle contains a large amount of T lymphocytes and transports immunoglobulins and cytokines. Continuous fluid leakage ends up impacting both the primary response^[44]^ and the humoral response to pathogens^[33]^. Besides, proper gradients guide proteins, peptides, macromolecules, nutrients, cells, and chemokines’ migration to the tissues, establishing the correct direction of interstitial-lymphatic capillaries flow. Therefore, chyle depletion will impair patients’ capacity to combat pathogens and regulate inflammation^[45]^. Besides, chyle also contains fat-soluble vitamins, proteins, electrolytes, and water, and consequently, chylothorax leads to hyponatremia, hypokalemia, and acidosis. The caloric loss in chyle pleural effusion rapidly induces severe protein-calorie malnutrition^[46]^.

Of patients undergoing NMPC for chylothorax, 37.1% will fail and require reintervention to obliterate the thoracic duct. The video-assisted thoracic duct ligation is probably the most applied reintervention technique^[23,27,30,31,33,34]^. During reoperations, one of the main difficulties is to find the site of lymphatic duct injury. Delayed intervention may create a field with intense inflammatory adherences, making it difficult to spot the site of injury. The administration of an oral cream containing long-chain triglycerides before surgery may help to find the spot of chyle leakage in the lymphatic duct^[14,27,29]^. Another alternative to obliterating thoracic duct systems is with interventional radiology. Lymphangiography is used to find the leak spot with subsequent embolization^[11]^, reducing the chyle drainage^[47]^.

Prolonged fasting with TPN aims to reduce the amount of chyle produced, helping recover the ruptured duct^[14]^. Parenteral nutrition has some inherent risks that should be taken into accounts, such as catheter-related bloodstream infections, venous thrombosis, and integrity loss of the gastrointestinal mucosa^[48]^. The central line complications may contribute to the high expected morbidity in NMPC. The compromised immunological status in chylothorax patients associated with the risk for bloodstream infection raises their mortality risks.

To reduce the risk of central line-associated bloodstream infections and other central line-associated complications, an alternative within the NMPC strategies is the MCT diet. By replacing the long-chain triglycerides for MCT supplementation, the amount of chyle produced would be reduced and, consequently, the loss of fluid and nutrients from the chylothorax^[29]^. MCT is absorbed directly into the blood, avoiding the overload of the lymphatic system. MCTs are easily ingested, rapidly absorbed, and readily metabolized directly into the portal venous system by passing the thoracic duct lymphatic system^[49]^. However, either by TPN or MCT therapy, it is expected to take a prolonged time for the injured lymphatic system to heal, imposing a prolonged time of thoracic tube usage, prolonged hospital stay, and increased hospital resources usage and inherent costs. Unlike blood vessels, chyle lacks coagulation factors and platelets, explaining the long time for the spontaneously leak flow reduction^[50]^.

Long-term chest tube use generates additional risks. Patients with prolonged use of chest tubes will face breath discomfort and higher demand for analgesics. The chest tube may also impair rib cage expansion, leading these patients to atelectasis, pleural effusion, and pneumonia^[51]^. Tube displacement, with subsequent emphysema and pneumothorax, may also occur, contributing to the increased risk of morbidity and mortality for patients^[52,53,54]^.

This systematic review presents the current evidence for chylothorax nonoperative management. Knowing the expected outcomes for nonoperative management, as shown in this meta-analysis, caregivers are able to expand their knowledge about this matter to make the best decisions for their patients. The poor outcomes of this strategy point that early reoperation may be an interesting alternative for chylothorax after cardiothoracic surgery.

### Limitations

The present study has some limitations. The concept of chylothorax is not homogeneous across the studies, with different definitions. The nonoperative methods for treating chylothorax are also variable across the studies, comprising different types of nutrition and time to decide to perform the reintervention. In addition, it must be considered that a chylothorax is a rare event and that the available studies do not have a large sample size to determine the level of evidence in this theme. The findings of the present study outlined the need for future controlled trials that compare nonoperative methods with early reoperation to verify the best treatment option for chylothorax after cardiothoracic surgery.

## CONCLUSION

Nonoperative treatment for chylothorax after cardiothoracic procedures has significant hospital stay, morbidity, mortality, and reoperation rates.

## Abbreviations, Acronyms & Symbols

CI: Confidence interval
GRADE: Grading of Recommendations, Assessment, Development and Evaluations
LOS: Length of hospital stay
MCT: Medium-chain triglycerides
NMPC: Nonoperative management of postoperative chylothorax
PICO: Patient, intervention, comparison, or outcome
ROBINS-I: Patient, intervention, comparison, or outcome
SD: Standart devlation
TPN: Total parenteral nutrition
